# Sex differences in invasive pneumococcal disease and the impact of pneumococcal conjugate vaccination in the Netherlands, 2004 to 2015

**DOI:** 10.2807/1560-7917.ES.2017.22.10.30481

**Published:** 2017-03-09

**Authors:** Gertjan H J Wagenvoort, Elisabeth A M Sanders, Bart J Vlaminckx, Hester E de Melker, Arie van der Ende, Mirjam J Knol

**Affiliations:** 1 Department of Medical Microbiology and Immunology, St. Antonius Hospital, Nieuwegein, the Netherlands; 2 Centre for Infectious Disease Control Netherlands (CIb), National Institute for Public Health and the Environment (RIVM), Bilthoven, the Netherlands; 3 Department of Immunology and Infectious diseases, University Medical Center Utrecht, Wilhelmina Children’s Hospital, Utrecht, the Netherlands; 4 Department of Medical Microbiology and the Netherlands Reference Laboratory for Bacterial Meningitis, Academic Medical Center, Amsterdam, the Netherlands

**Keywords:** *Streptococcus pneumoniae*, pneumococcal conjugate vaccine, invasive pneumococcal disease, sentinel surveillance, sex, epidemiology

## Abstract

Implementation of pneumococcal conjugate vaccines in the Netherlands (PCV7 in 2006 and PCV10 in 2011) for infants caused a shift in serotypes in invasive pneumococcal disease (IPD). We explored sex differences in serotype-specific IPD incidence before and after vaccine introduction. Incidences in the pre-PCV7 (June 2004–May 2006), post-PCV7 (June 2008–May 2011) and post-PCV10 period (June 2013–May 2015), stratified by age, were compared. Incidence was higher in men for all age groups (overall in men: 16.7, 15.5 and 14.4/100,000 and women: 15.4, 13.6 and 13.9/100,000 pre-PCV7, post-PCV7 and post-PCV10, respectively), except for 20–39 year-olds after PCV7 and 40–64 year-olds after PCV10 introduction. After PCV7 and PCV10 introduction, the overall IPD incidence decreased in men aged 20–39 years (from 5.3 pre-PCV7 to 4.7 and 2.6/100,000 post-PCV7 and post-PCV10, respectively), whereas it showed a temporary increase in women (from 3.9/100,000 pre-PCV7 to 5.0/100,000 post-PCV7 and back to 4.0/100,000 post-PCV10) due to replacement disease. PCV10 herd effects were observed throughout, but in women older than 40 years, a significant increase in non-PCV10 serotype offset a decrease in overall IPD incidence. Ongoing surveillance of IPD incidence by sex is important to evaluate the long-term effects of PCV implementation.

## Introduction

Sex differences play an important role in clinical disease susceptibility and outcome. In infectious diseases, the burden of bacterial, fungal, parasitic and viral disease is generally higher in men than in women [1-3]. However, with the exception of urinary tract infections, sex differences are often neglected in surveillance reports [4,5]. Also for *Streptococcus pneumoniae*, a frequent coloniser of the nasopharynx and cause of severe infections, the observed incidences of pneumococcal pneumonia and invasive pneumococcal disease (IPD) have been higher in men [6-8]. Young children have the highest pneumococcal carriage rates and are the key transmitters of *S. pneumoniae* in the population. However, no systematic age-specific differences in asymptomatic pneumococcal nasopharyngeal carriage rates have been observed between boys and girls [9-11]. Pneumococcal conjugate vaccination (PCV) has led to eradication of vaccine serotype carriage but immediate replacement by non-vaccine serotypes with a modest reduction in overall pneumococcal carriage in children [12,13].

In many countries, extensive IPD surveillance programmes were implemented before and after introduction of PCV in childhood immunisation programmes [14-17]. Although many surveillance reports described the impact of the first licensed 7-valent PCV (PCV7) on (serotype-specific) IPD incidences in different age groups [17], only limited data have been reported by sex [18-20]. In the Netherlands, PCV7 has been introduced for infants born after 1 April 2006 (in a 3 + 1 schedule) with a coverage of 94–95% since its introduction [21] and has led to a shift from vaccine to non-vaccine serotypes causing IPD (‘replacement disease’) in all age groups. In children eligible for vaccination, the decline in vaccine serotype IPD was strongest, but overall IPD incidence decreased due to herd protection in most age groups, in particular in persons 65 years and older [16,17].

Differences in the impact of pneumococcal conjugate vaccines between men and women do occur. Hak et al. reported an increase in the incidence of pneumococcal pneumonia in mothers of young infants after introduction of PCV7 in the United Kingdom (UK) [22]. In the Netherlands, a significant increase in IPD incidence was observed in middle-aged women 2–4 years after introduction of PCV7, mainly due to the emergence of serotype 1, which was not observed in men in the same age group [23]. Whether this was due to non-PCV7 serotype replacement or to a secular trend needs to be established because serotype 1 is a naturally fluctuating serotype [24]. In May 2011, the 10-valent pneumococcal conjugate vaccine (PCV10) including serotype 1 was introduced for Dutch infants.

The objective of this study was to explore differences in IPD incidence between men and women before and after PCV7 and PCV10 introduction by using national surveillance data up to May 2015.

## Methods

### Study population and data collection

The Dutch pneumococcal surveillance is based on data from nine sentinel laboratories covering different regions of the Netherlands and ca 25% of the Dutch population (ca 4.2 million inhabitants, including 2.07 million men and 2.11 million women). The participant laboratories, selected for geographic location and reliability for submitting isolates, have not changed over time during the study period [14,16]. In addition, we have no indication that surveillance sensitivity has changed over the years. Pneumococcal isolates of all IPD patients, defined as patients with *S. pneumoniae* isolated from blood or cerebrospinal fluid (CSF), were submitted to the Netherlands Reference Laboratory for Bacterial Meningitis (NRLBM) for serotyping by co-agglutination and capsular swelling (Quellung reaction) using specific antisera (Statens Serum Institute, Denmark). Pneumococcal serotypes and demographic data including age and sex were available for IPD cases from June 2004 up to May 2015.

In addition, clinical information including clinical syndromes (categorised as (i) meningitis, (ii) invasive pneumonia (without meningitis), (iii) bacteraemia without focus and (iv) bacteraemia with other focus (without meningitis or invasive pneumonia)), clinical outcome (death in hospital and/or death within 30 days after first reported blood/CSF culture positive for *S. pneumoniae),* admission to an intensive care unit (ICU), and presence of underlying conditions (immunocompromising conditions and other comorbidities) were retrospectively extracted for IPD patients from June 2004 up to May 2012 from hospital medical records as described [15,16,25]. Clinical data from the post-PCV10 period (1 June 2013 to 31 May 2015) were not available.

### Data analysis

We assessed sex-specific IPD incidences during a pre-PCV7 (1 June 2004 to 31 May 2006), post-PCV7 (1 June 2008 to 31 May 2011) and post-PCV10 period (1 June 2013 to 31 May 2015) and calculated the female/male (F/M) incidence ratio. The first two years after introduction of PCV7 and PCV10 (1 June 2006 to 31 May 2008 and 1 June 2011 to 31 May 2013) were regarded as transition period and not included. 

Changes in IPD incidences, comparing post-PCV7 to pre-PCV7 and post-PCV10 to post-PCV7 were assessed for men and women separately by calculating relative risks (RR). To investigate sex differences in direct and indirect effects (i.e. changes in IPD incidences) after PCV7 or PCV10 introduction, the interaction between sex and change in IPD incidence (RR post-PCV7 to pre-PCV7 or RR post-PCV10 to post-PCV7, respectively) was assessed by calculating the F/M risk ratio of RRs (dividing the RR in women by the RR in men). 

Likewise, sex-specific differences in the proportion of clinical syndromes, in clinical outcome and in underlying conditions were assessed in the pre-PCV7 and post-PCV7 period (F/M ratio of proportions). Also changes in the proportion of clinical syndromes, in clinical outcome and in underlying conditions after PCV7 were assessed for men and women separately (RR post-PCV7 to pre-PCV7). The interaction between sex and change in proportions was assessed by calculating the F/M risk ratio of RRs. Cases without clinical data were excluded from these analyses.

We further explored if there was a sex-specific preference in certain serotypes causing IPD, defined as an intrinsic and stable factor influencing the occurrence of a serotype that would potentially explain differences in overall IPD susceptibility between sexes. Potential sex-specific preferences in serotypes causing IPD were assessed by calculating the serotype-specific F/M ratio. We used all data from 2004 to 2015 (including the transition years) from patients aged 5 years and older (n = 6,628) to account for changes over time, without any exclusion. Younger children (n = 276) were not taken into account for this analysis because the numbers of IPD per serotype were too small to analyse them as a separate group. The serotype-specific F/M ratios were compared with the average F/M ratio (dividing the total number of cases in women by the total number of cases in men across all serotypes in patients aged 5 years and older) using Fisher's exact test (SPSS version 22). A p value of < 0.05 was considered statistically significant.

Analyses were stratified by age group (0–4, 5–19, 20–39, 40–64 and ≥ 65 years-old). Serotypes were divided in PCV7 serotypes (i.e. serotype 4, 6B, 9V, 14, 18C, 19F and 23F), additional PCV10 serotypes (‘PCV10 extra’: i.e. serotype 1, 5 and 7F) and non-PCV7 or non-PCV10 serotypes (all serotypes not included in PCV7 or PCV10, respectively). Also, as a sensitivity analysis, serotype 6A was included as a PCV7 serotype and results remained similar (data not shown).

Incidences were calculated as the number of cases per 100,000 persons per year. National incidences were calculated by dividing the population number for each year (StatLine Statistics, the Netherlands) by 4 reflecting the coverage of the sentinel surveillance laboratories (25% of the Dutch population). Differences in incidences and proportions were tested with chi-squared or Fisher’s exact test, as appropriate, and for F/M incidence ratio, F/M ratio of proportions, serotype-specific F/M ratio, relative risks and F/M risk ratio of RRs, 95% confidence intervals (CI) were calculated using 2 × 2 tables (z-distribution) [26].

## Results

Overall, 6,906 patients were affected by IPD in the period from 1 June 2004 to 31 May 2015; 3,592 males and 3,314 females, including 169 males and 107 females younger than 5 years. For the pre- and post-PCV periods (without the transition years included), overall 4,303 patients had IPD; 2,227 males and 2,076 females, including 106 males and 79 females younger than 5 years.

### Incidence of invasive pneumococcal disease pre- and post-PCV7/10

**Figure 1:**
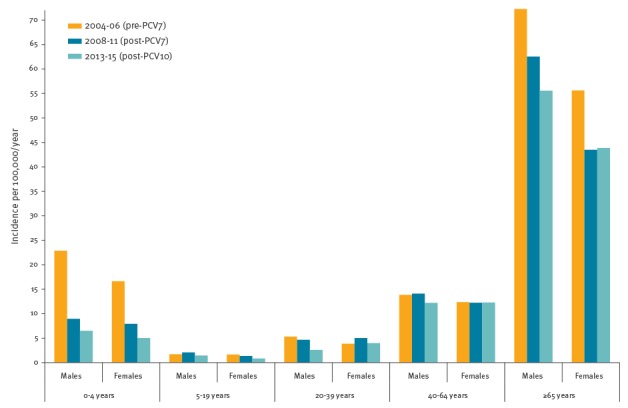
Age-specific incidence of invasive pneumococcal disease in men and women in the pre- and post-PCV periods, the Netherlands, 2004–15 (n = 4,303)

**Figure 2:**
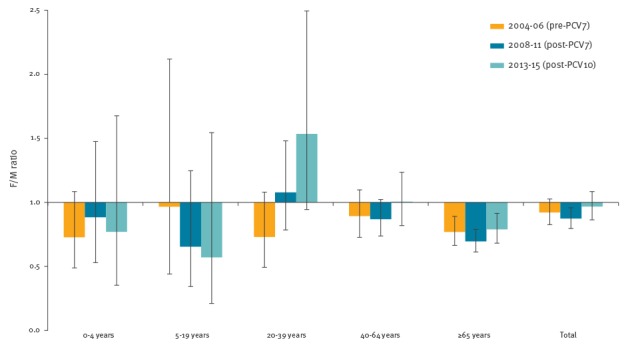
Age-specific female/male incidence ratio of invasive pneumococcal disease in the pre- and post-PCV periods, the Netherlands, 2004–15 (n = 4,303)

Figure 1 shows the age and sex-specific IPD incidence in the pre-PCV7, post-PCV7 and post-PCV10 period. The IPD incidence in men was higher than in women for all age groups, except for the 20–39 year-olds in both post-PCV periods and the 40–64 year-olds in the post-PCV10 period. The largest difference in IPD incidence between men and women (in absolute numbers, Figure 1) was observed in those at highest risk, i.e. children younger than 5 years and persons 65 years and older, but the F/M ratio was only significant for those 65 years and older (Figure 2, F/M incidence ratio).

PCV: pneumococcal conjugate vaccine.

Study period: pre-PCV7 (June 2004–May 2006) post-PCV7 (June 2008–May 2011) and post-PCV10 (June 2013–May 2015).

F/M: female/male; PCV: pneumococcal conjugate vaccine.

Study period: pre-PCV7: June 2004–May 2006; post-PCV7: June 2008–May 2011; post-PCV10: June 2013–May 2015.

Black bars indicate 95% confidence intervals.

The Table presents IPD incidences (and absolute number of cases) for male and female IPD patients in the pre-PCV7, post-PCV7 and post-PCV10 periods and the change in IPD incidence for men and women separately (RR comparing post-PCV7 to pre-PCV7 and RR post-PCV10 to post-PCV7). In addition, we used the F/M risk ratio of RRs (post-PCV7 vs pre-PCV7 and post-PCV10 vs post-PCV10) to investigate sex differences in direct and indirect effects. Comparing post-PCV7 to pre-PCV7, there was a significant decrease in overall IPD incidence for children younger than 5 years and and people 65 years and older in both sexes (Table: in those younger than 5 years, the male RR was 0.39 (95% CI: 0.25–0.60) and the female RR was 0.48 (95% CI: 0.29–0.77), and in those 65 years and older, the male RR was 0.87 (95% CI: 0.76–0.99) and the female RR was 0.78 (95% CI: 0.68–0.90). The overall reduction (F/M risk ratio of RRs post-PCV7 vs pre-PCV7) in IPD was not significantly different between men and women of these age groups (Table).

**Table:** IPD incidences for male and female invasive pneumococcal disease patients in the pre-PCV7, post-PCV7 and post-PCV10 period, the Netherlands, 2004–15 (n = 4,303)

Age group	Pre-PCV7 (2004–06)		Post-PCV7 (2008–11)		Post-PCV10 (2013–15)		Men post-PCV7 vs pre-PCV7	Women post-PCV7 vs pre-PCV7	F/M risk ratio RRs post-PCV7 vs pre-PCV7	Men post-PCV10 vs post-PCV7	Women post-PCV10 vs post-PCV7	F/M risk ratio RRs post-PCV10-vs post-PCV7
	Male	Female	Male	Female	Male	Female						
	Incidence (n)	Incidence (n)	Incidence (n)	Incidence (n)	Incidence (n)	Incidence (n)	RR (95% CI)^a^	RR (95% CI)^b^	Risk ratio (95% CI)^c^	RR (95% CI)^d^	RR (95% CI)^e^	Risk ratio (95% CI)^f^
**All ages**	16.7 (674)	15.4 (634)	15.5 (953)	13.6 (850)	14.4 (599)	13.9 (591)	0.93 (0.84–1.03)	***0.88*** ***(0.80–0.98)***	0.95 (0.82–1.09)	0.92 (0.83–1.02)	1.02 (0.92–1.14)	1.11 (0.96–1.28)
PCV7	8.0 (321)	6.9 (286)	3.2 (194)	2.9 (184)	0.8 (32)	0.6 (24)	***0.40*** ***(0.33–0.47)***	***0.42*** ***(0.35–0.51)***	1.06 (0.82–1.38)	***0.24*** ***(0.17–0.35)***	***0.19*** ***(0.13–0.29)***	0.79 (0.45–1.40)
PCV10 extra	3.3 (132)	2.7 (111)	3.6 (221)	3.7 (229)	2.5 (105)	2.4 (100)	1.10 (0.89–1.37)	***1.36*** ***(1.08–1.70)***	1.23 (0.90–1.69)	***0.70*** ***(0.55–0.88)***	***0.64*** ***(0.51–0.81)***	0.92 (0.66–1.28)
non-PCV7	8.8 (353)	8.5 (348)	12.4 (759)	10.6 (666)	13.6 (567)	13.3 (567)	***1.41*** ***(1.25–1.60)***	***1.26*** ***(1.11–1.43)***	0.89 (0.74–1.07)	1.10 (0.99–1.23)	***1.25*** ***(1.12–1.40)***	1.14 (0.98–1.33)
non-PCV10	5.5 (221)	5.8 (237)	8.8 (538)	7.0 (437)	11.1 (462)	11 (467)	***1.60*** ***(1.37–1.87)***	***1.21*** ***(1.04–1.42)***	***0.76*** ***(0.61–0.95)***	***1.26*** ***(1.12–1.43)***	***1.58*** ***(1.38–1.79)***	***1.25*** ***(1.04–1.49)***
**< 5 years**	22.9 (59)	16.6 (41)	9.0 (32)	7.9 (27)	6.5 (15)	5.0 (11)	***0.39*** ***(0.25–0.60)***	***0.48*** ***(0.29–0.77)***	1.21 (0.63–2.32)	0.73 (0.39–1.34)	0.63 (0.31–1.28)	0.87 (0.34–2.21)
PCV7	14.7 (38)	12.2 (30)	0.6 (2)	0.6 (2)	0.4 (1)	0.0 (0)	***0.04*** ***(0.01–0.16)***	***0.05*** ***(0.01–0.20)***	1.27 (0.17–9.53)	0.78 (0.07–8.55)	NA	NA
PCV10 extra	3.5 (9)	1.2 (3)	2.5 (9)	2.6 (9)	0.9 (2)	0.5 (1)	0.72 (0.29–1.82)	2.17 (0.59–8.01)	3.00 (0.61–14.87)	0.34 (0.07–1.59)	0.17 (0.02–1.36)	0.50 (0.04–6.56)
non-PCV7	8.1 (21)	4.5 (11)	8.4 (30)	7.3 (25)	6.1 (14)	5.0 (11)	1.03 (0.59–1.80)	1.64 (0.81–3.34)	1.59 (0.65–3.92)	0.72 (0.38–1.36)	0.68 (0.34–1.39)	0.94 (0.36–2.45)
non-PCV10	4.6 (12)	3.2 (8)	5.9 (21)	4.7 (16)	5.2 (12)	4.6 (10)	1.26 (0.62–2.57)	1.45 (0.62–3.38)	1.14 (0.38–3.45)	0.89 (0.44–1.80)	0.97 (0.44–2.14)	1.10 (0.38–3.17)
**5–19 years**	1.7 (13)	1.7 (12)	2.1 (24)	1.4 (15)	1.5 (11)	0.8 (6)	1.22 (0.62–2.40)	0.83 (0.39–1.77)	0.68 (0.25–1.87)	0.70 (0.34–1.43)	0.61 (0.24–1.57)	0.87 (0.27–2.86)
PCV7	0.8 (6)	0.7 (5)	0.3 (4)	0.5 (5)	0.3 (2)	0.0 (0)	0.44 (0.12–1.56)	0.66 (0.19–2.28)	1.50 (0.26–8.81)	0.76 (0.14–4.16)	NA	NA
PCV10 extra	0.4 (3)	0.4 (3)	1.1 (13)	0.6 (7)	0.4 (3)	0.4 (3)	2.87 (0.82–10.06)	1.54 (0.40–5.97)	0.54 (0.09–3.41)	0.35 (0.1–1.24)	0.65 (0.17–2.53)	1.86 (0.29–11.76)
non-PCV7	0.9 (7)	1.0 (7)	1.7 (20)	0.9 (10)	1.2 (9)	0.8 (6)	1.89 (0.80–4.47)	0.94 (0.36–2.48)	0.50 (0.14–1.82)	0.69 (0.31–1.51)	0.92 (0.33–2.52)	1.33 (0.37–4.81)
non-PCV10	0.5 (4)	0.6 (4)	0.6 (7)	0.3 (3)	0.8 (6)	0.4 (3)	1.16 (0.34–3.96)	0.5 (0.11–2.22)	0.43 (0.06–2.97)	1.31 (0.44–3.89)	1.53 (0.31–7.56)	1.17 (0.17–8.09)
**20–39 years**	5.3 (60)	3.9 (43)	4.7 (74)	5.0 (79)	2.6 (27)	4.0 (41)	0.88 (0.62–1.23)	1.29 (0.89–1.88)	1.48 (0.89–2.44)	***0.56*** ***(0.36–0.87)***	0.80 (0.55–1.16)	1.42 (0.80–2.54)
PCV7	1.7 (19)	1.7 (19)	0.9 (15)	1.3 (20)	0.2 (2)	0.1 (1)	0.56 (0.29–1.10)	0.74 (0.40–1.39)	1.32 (0.53–3.33)	***0.20*** ***(0.05–0.89)***	***0.08*** ***(0.01–0.57)***	0.38 (0.03–4.54)
PCV10 extra	2.3 (26)	1.2 (13)	2.0 (31)	2.4 (38)	1.2 (12)	1.4 (14)	0.85 (0.50–1.43)	**2.06** **(1.10–3.87)**	***2.43*** ***(1.07–5.50)***	0.59 (0.3–1.16)	0.57 (0.31–1.04)	0.95 (0.39–2.35)
non-PCV7	3.6 (41)	2.2 (24)	3.7 (59)	3.7 (59)	2.4 (25)	3.9 (40)	1.02 (0.69–1.52)	**1.73** **(1.08–2.78)**	1.69 (0.91–3.15)	0.65 (0.41–1.04)	1.04 (0.70–1.55)	1.60 (0.86–2.97)
non-PCV10	1.3 (15)	1.0 (11)	1.8 (28)	1.3 (21)	1.3 (13)	2.5 (26)	1.33 (0.71–2.48)	1.34 (0.65–2.79)	1.01 (0.39–2.65)	0.71 (0.37–1.37)	***1.90*** ***(1.07–3.38)***	***2.67*** ***(1.11–6.39)***
**40–64 years**	13.8 (194)	12.4 (170)	14.1 (313)	12.2 (268)	12.2 (182)	12.3 (182)	1.02 (0.85–1.22)	0.99 (0.82–1.20)	0.97 (0.75–1.26)	0.87 (0.72–1.04)	1.00 (0.83–1.21)	1.16 (0.89–1.51)
PCV7	5.9 (82)	4.6 (63)	2.9 (64)	2.4 (52)	0.5 (7)	0.3 (5)	***0.49*** ***(0.36–0.68)***	***0.52*** ***(0.36–0.75)***	1.05 (0.64–1.72)	***0.16*** ***(0.07–0.36)***	***0.14*** ***(0.06–0.36)***	0.87 (0.26–2.91)
PCV10 extra	2.6 (37)	2.5 (35)	3.6 (80)	3.8 (83)	2.4 (36)	2.7 (40)	1.37 (0.92–2.02)	***1.49*** ***(1.00–2.21)***	1.09 (0.63–1.90)	***0.67*** ***(0.45–0.99)***	0.71 (0.49–1.04)	1.06 (0.62–1.83)
non-PCV7	8.0 (112)	7.8 (107)	11.2 (249)	9.9 (216)	11.7 (175)	11.9 (177)	***1.40*** ***(1.12–1.75)***	***1.27*** ***(1.01–1.60)***	0.90 (0.65–1.25)	1.05 (0.86–1.27)	1.21 (0.99–1.48)	1.16 (0.88–1.53)
non-PCV10	5.4 (75)	5.2 (72)	7.6 (169)	6.1 (133)	9.3 (139)	9.2 (137)	***1.42*** ***(1.08–1.87)***	1.16 (0.87–1.55)	0.82 (0.55–1.21)	1.22 (0.98–1.53)	**1.52** **(1.20–1.93)**	1.24 (0.90–1.72)
**≥ 65 years**	72.3 (348)	55.6 (368)	62.5 (510)	43.5 (461)	55.6 (364)	43.9 (351)	***0.87*** ***(0.76–0.99)***	***0.78*** ***(0.68–0.90)***	0.90 (0.75–1.10)	0.89 (0.78–1.02)	1.01 (0.88–1.16)	1.13 (0.94–1.38)
PCV7	36.3 (175)	25.5 (169)	13.4 (109)	9.8 (104)	3.1 (20)	2.3 (18)	***0.37*** ***(0.29–0.47)***	***0.38*** ***(0.30–0.49)***	1.04 (0.74–1.47)	***0.23*** ***(0.14–0.37)***	***0.23*** ***(0.14–0.38)***	1.00 (0.50–2.00)
PCV10 extra	11.8 (57)	8.6 (57)	10.8 (88)	8.7 (92)	7.9 (52)	5.3 (42)	0.91 (0.65–1.27)	1.01 (0.72–1.40)	1.11 (0.69–1.77)	0.74 (0.52–1.04)	***0.60*** ***(0.42–0.87)***	0.82 (0.50–1.36)
non-PCV7	35.9 (173)	30.1 (199)	49.2 (401)	33.7 (357)	52.5 (344)	41.6 (333)	***1.37*** ***(1.15–1.64)***	1.12 (0.94–1.33)	0.82 (0.64–1.05)	1.07 (0.92–1.23)	***1.24*** ***(1.06–1.43)***	1.16 (0.94–1.42)
non-PCV10	24.1 (116)	21.5 (142)	38.4 (313)	25.0 (265)	44.6 (292)	36.4 (291)	***1.59*** ***(1.29–1.97)***	1.17 (0.95–1.43)	***0.73*** ***(0.54–0.98***)	1.16 (0.99–1.36)	***1.45*** ***(1.23–1.72)***	1.25 (0.99–1.58)

CI: confidence interval; F/M: female/male; PCV7: 7-valent pneumococcal conjugate vaccine; PCV10: 10-valent pneumococcal conjugate vaccine; RR: relative risk; PCV10 extra: serotypes 1, 5, 7F.

Difference in proportions were tested with chi-squared test, and RR and 95% CI were calculated. Incidences are shown as number of cases/100,000/year). Analyses were stratified by age group (< 5, 5–19, 20–39, 40–64, ≥ 65 years). Numbers in italics indicates a significant difference.

Study period: pre-PCV7: June 2004–May 2006; post-PCV7: June 2008–May 2011; post-PCV10: June 2013–May 2015.

**Figure 3:**
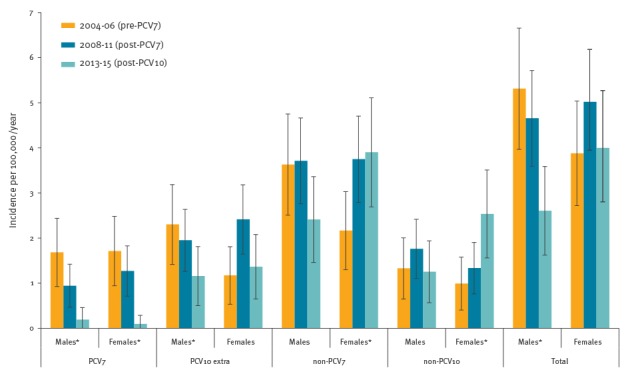
Incidences of invasive pneumococcal disease per serotype group in 20–39 year-old men and women pre-PCV7, post-PCV7 and post-PCV10, the Netherlands, 2004–15 (n = 324)

IPD: invasive pneumococcal disease; PCV: pneumococcal conjugate vaccine.

Also in other age groups, there was no significant interaction between change in IPD incidence and sex comparing post-PCV7 to pre-PCV7. However, in the 20–39 year-olds, the F/M risk ratio of RRs for PCV10 serotype IPD was 2.43 (95% CI: 1.07–5.50), owing to a significant increase in PCV10 serotype IPD in women (RR = 2.06; 95% CI: 1.10–3.87) and a non-significant reduction in men (RR = 0.85; 95% CI: 0.50–1.43), which indicated a statistically significant difference in replacement disease by PCV10 serotypes (mainly 1 and 7F, data not shown). As a result, women showed a non-significant increase in IPD incidence comparing the pre-PCV7 to post-PCV7 (from 3.9/100,000 to 5.0/100,000) period, whereas the incidence decreased in men of the same age (from 5.3/100,000 to 4.7/100,000, Figure 3). In 40–64 year-olds of both sexes, IPD incidence remained stable after PCV7 introduction.

Study period: pre-PCV7: June 2004–May 2006; post-PCV7: June 2008–May 2011; post-PCV10: June 2013–May 2015, Black bars indicate 95% confidence intervals.

* Indicates a significant difference (p value < 0.05) in IPD incidence comparing post-PCV10 to pre-PCV7.

After PCV10 introduction, IPD caused by the additional PCV10 serotypes decreased in all age groups and in both sexes, suggesting PCV10 herd protection, although this was only significant for men aged 40–64 (RR = 0.67; 95% CI: 0.45–0.99) and women 65 years and older (RR = 0.60; 95% CI: 0.42–0.87, Table). In addition, PCV7 serotype IPD continued to decrease. These on-going effects of herd protection against PCV7 and recently introduced PCV10 resulted in a non-significant decline in overall IPD incidence for all except women aged 40 and older. In women 40 years and older, PCV7/10 herd protection was offset by a significant increase in non-PCV10 serotype IPD, which was not observed in men, a group where IPD incidence declined. As a result, IPD incidence in the 40–64 year-olds became similar in women and men (12.3/100,000 vs 12.2/100,000). However, we did not observe a significantly different change in overall IPD incidence between men and women in this age group (F/M risk ratio of RRs post-PCV10 vs post-PCV7, Table). Also in other age groups, there was no significant interaction between change in overall IPD incidence and sex comparing post-PCV10 to post-PCV7. After PCV10 introduction, non-PCV10 serotype IPD incidence in 20–39 year-old women increased (RR = 1.90; 95% CI: 1.07–3.38), whereas it decreased in men (RR = 0.71; 95% CI: 0.37–1.37, F/M risk ratio of RRs = 2.67; 95% CI: 1.11–6.39).

### Clinical syndromes, outcomes and underlying conditions pre- and post-PCV7

Comparing post-PCV7 to pre-PCV7, no major differences were observed between sexes regarding shifts in the proportion of different clinical syndromes, outcomes and underlying conditions. However, some significant differences within the pre- and/or post-PCV7 periods, and small but significantly different shifts in proportions were observed between male and female IPD patients.

#### Clinical syndromes

In the pre-PCV7 period, the overall proportion (without stratification for age) of IPD patients with invasive pneumonia was significantly higher in male than female patients (76 vs 71%; F/M ratio of proportions: RR = 0.93; 95% CI: 0.87–0.98). After stratification for age, this remained significant for the 20–39 year-olds and those 65 years and older. In the post-PCV7 period (June 2008–May 2011), the distribution of clinical syndromes in all IPD patients was not significantly different between male and female patients. However, in male IPD patients aged 5–19 years, the proportion of pneumonia was significantly higher than in female patients (80 vs 40%: F/M ratio of proportions: RR = 0.50; 95% CI: 0.26–0.96), whereas for 20–39 year-old men, meningitis was significantly more common (20 vs 8%; F/M ratio of proportions: RR = 0.39; 95% CI: 0.16–0.96). The changes in distribution of clinical syndromes after PCV7 introduction (F/M risk ratio of RRs) were not significantly different between male and female patients except in 20–39 year-olds, in whom the increased proportion of pneumonia in female patients (from 68 to 77%) differed significantly from the decreased proportion (from 88 to 73%) in male patients (F/M risk ratio of RRs = 1.37; 95% CI: 1.01–1.86).

#### Outcomes

In the pre- and post-PCV7 period, the overall case fatality and the proportion of ICU admissions (without stratification for age) were not significantly different between male and female IPD patients. The decline in case fatality after PCV7 introduction did not differ significantly between male (from 15 to 12%) and female patients (from 18 to 11%; F/M risk ratio of RRs = 0.79; 95% CI: 0.54–1.13). Nor were changes in case fatality and proportion of ICU admissions significantly different between male and female patients after stratification for age.

#### Underlying conditions

In the pre- and post-PCV7 period, the overall proportion (without stratification for age) of male IPD patients with an immunocompromising condition was significantly higher compared with that of female patients, with 21 vs 16% pre-PCV7 (F/M ratio of proportions = 0.78; 95% CI: 0.61–0.99) and 21 vs 13% post-PCV7 (F/M ratio of proportions = 0.64; 95% CI: 0.51–0.79), respectively. After stratification for age, this remained significant for patients 65 years and older. Also the change in the overall proportion of patients with an immunocompromising condition after PCV7 introduction was significantly different between male and female patients (F/M risk ratio of RRs = 0.82; 95% CI: 0.67–0.99), indicating a significantly higher decrease in women compared with the stable proportion in men. However, after stratification for age, there were no significant differences in changes in immunocompromising conditions between male and female patients following PCV7 introduction.

The overall proportion of patients with any comorbidity was higher in male IPD patients than in female patients, with 76 vs 74% pre-PCV7 (F/M ratio of proportions = 0.98; 95% CI: 0.92–1.05) and 77 vs 72% post-PCV7 (F/M ratio of proportions = 0.94; 95% CI: 0.89–0.99). After stratification for age, this was significant for patients 65 years and older pre-PCV7 and for 40–64 year-old patients post-PCV7. The overall change in the proportion of patients with any comorbidity following PCV7 introduction was not significantly different between male and female patients (F/M risk ratio of RRs = 0.96; 95% CI: 0.88–1.04) with or without stratification for age.

### Serotype-specific female/male ratio for invasive pneumococcal disease

**Figure 4:**
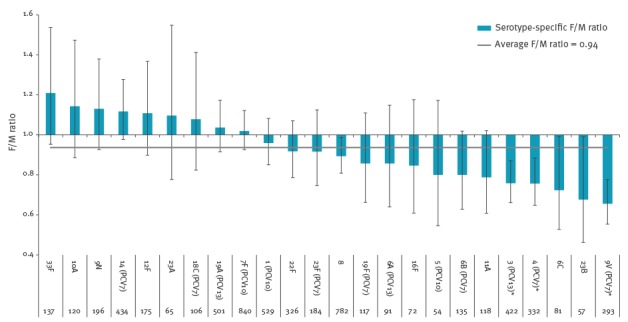
Serotype-specific female/male ratio for causing invasive pneumococcal disease in patients 5 years and older, the Netherlands, 2004–15 (n = 6,628)

Figure 4 shows the serotype-specific F/M ratio. Overall, 6,628 patients aged 5 years and older (3,422 males, 3,206 females) were affected by IPD in the period from 1 June 2004 to 31 May 2015. The average F/M ratio across all serotypes was 0.94. Only serotypes 3, 4 and 9V were significantly associated with male sex (F/M ratio = 0.76; p = 0.026, F/M ratio = 0.76; p = 0.047 and F/M ratio = 0.66; p = 0.002, respectively).

F/M: female/male; IPD: invasive pneumococcal disease; PCV: pneumococcal vaccine.

All (additional) serotypes included in the 7, 10 and (not introduced) 13-valent PCV (‘PCV13 extra’: i.e. serotype 3, 6A and 19A) are shown. Of the non-vaccine serotypes, all serotypes with more than 50 cases during the entire study period are shown. The horizontal line represents the average F/M ratio across all serotypes (0.94). The numbers on the x axis represent the total number of isolates per serotype. Black bars indicate 95% confidence intervals.

* Indicates a significant difference (p value < 0.05) between the serotype-specific F/M ratio compared and the F/M ratio all other serotypes combined.

## Discussion

Our findings confirm the importance of sex as an epidemiological factor in IPD. We observed structurally higher IPD incidences in men in all age groups, with the exception of 20–39 year-olds after implementation of PCV7 and of 40–64 year-olds after implementation of PCV10. These observations illustrate a sex-specific differential impact of post-PCV dynamics.

The structural excess in IPD incidence in boys younger than 5 years has been attributed to anatomical or early hormonal differences predisposing to differences in immunity [27-29]. Likewise, the higher susceptibility for IPD in elderly men could be explained by sex-based inactivation of the X chromosome, resulting in differences in immunity [1,4,5]. Also a higher prevalence of underlying conditions in the male population such as chronic cardiovascular and renal disease, malignancies [30] or tobacco use is likely to play a role [31]. This is reflected in a significantly higher proportion of immunocompromising conditions in male IPD patients pre- and post-PCV7. We only found a serotype-specific preference to affect men and women differently in three of 24 serotypes; therefore an individual serotype-specific (or serotype-related) factor for explaining the difference in IPD susceptibility is unlikely.

In 20–39 year-olds, IPD incidence in women increased post-PCV7 and became higher than in men, in whom IPD incidence had decreased. This reflected a significant increase in IPD caused by the additional PCV10 serotypes (mainly serotypes 1 and 7F). Likewise, previous analysis of Dutch surveillance data 2–4 years after PCV7 introduction showed an increase in IPD incidence in women (20–44 years-old) caused by an increase in serotype 1 [23]. After PCV7 was replaced by PCV10, covering serotypes 1 and 7F, herd effects may have reduced the elevated burden of IPD in 20–39 year-old women. Indeed, herd protection of PCV10 was established, and overall IPD incidence as well as IPD caused by PCV10 serotypes decreased in both sexes. However, a significant increase in non-PCV10 type IPD was, again, exclusively observed in women and IPD incidence remained higher in women than in men post-PCV10. The increase in non-PCV10 type IPD (attributable to several serotypes) suggests a vaccine induced effect rather than natural fluctuation of a single serotype.

Also, in a recent study from the United States, IPD incidence was generally higher in male people, but after PCV13 introduction, IPD incidence among 18–39 year-old black women became slightly higher than in men [20]. Socially defined roles may explain this phenomenon. Women of childbearing age could be at increased risk for replacement disease because of close contact with PCV7/10-vaccinated children as was hypothesised in other studies [22,23]. In Scotland before PCV7 introduction, a higher IPD incidence was observed in 35–49 year-old women based on data from 1992 to 2007 [32]. Increased carriage of non-vaccine pneumococcal serotypes in parents of vaccinated children compared with parents of unvaccinated children has been well established [12,33]. Although these studies did not analyse men and women separately, the importance of sex is further supported by a study on pertussis, which showed a higher transmission rate between infants and mothers compared with fathers [34].

In other non-vaccinated age groups, PCV10 herd effects became apparent as well, but again with a sex-specific differential impact. In men 40 years and older, IPD incidence declined further during the post-PCV10 period, whereas herd effects in women of the same age groups were offset by a significant increase in IPD caused by non-PCV10 serotypes. This could suggest that for women 40 years and older, after an initial overall reduction in IPD due to PCV7 herd effects, a new plateau phase in IPD incidence has been reached using current vaccination strategies.

After introduction of PCV7, shifts in circulating serotypes were associated with significant changes in clinical outcome, such as a lower overall case fatality [25]. Our findings confirm that this is the case for both sexes at the time point 5 years after PCV7 introduction. Pre- and post-PCV7, the case fatality and ICU admission rates between male and female patients were not significantly different.

A limitation of our study is that it was an ecological study, so one should be cautious about interpreting findings as causally related to introduction of vaccination. In addition, not accounting for multiple testing in statistical analysis and some results being borderline significant, our results should be regarded as explorative analysis and need to be interpreted with caution. Furthermore, we had no information whether or not IPD patients were parents and/or had close contact with children and therefore could not further assess the proposed mechanism which could have resulted in the observed differences in 20–39 year-old patients. Nevertheless, our study provides important insights into structural sex differences in IPD incidence and different indirect effects after PCV7/10 introduction. This finding indicates that an intervention in a complex ecosystem can result in (temporary) changes in IPD dynamics but needs to be confirmed by others.

## Conclusion 

This study confirms the importance of sex in IPD incidence. We have shown that shifts in serotypes can cause increased IPD incidence rates. Although IPD surveillance studies have been performed in many countries, data on sex differences are scarce. We invite other investigators to stratify their pre- and post-pneumococcal vaccination IPD data by sex. Continued surveillance of IPD incidence and outcome by sex is important to evaluate the direct and indirect long-term effects of pneumococcal conjugate vaccination in the population.
